# Extracts of *Perilla frutescens* var. *Acuta* (Odash.) Kudo Leaves Have Antitumor Effects on Breast Cancer Cells by Suppressing YAP Activity

**DOI:** 10.1155/2021/5619761

**Published:** 2021-02-15

**Authors:** Cho-Long Kim, Yu-Su Shin, Sue-Hee Choi, Seroc Oh, Kyeongseob Kim, Han-Sol Jeong, Jung-Soon Mo

**Affiliations:** ^1^Department of Biomedical Sciences, Graduate School, Ajou University School of Medicine, Suwon 16499, Republic of Korea; ^2^Department of Ginseng and Medicinal Herb, National Institute of Horticultural and Herbal Science (NIHHS), Rural Development Administration (RDA), Wanju, Jeollabuk-Do 55365, Republic of Korea; ^3^Division of Applied Medicine, School of Korean Medicine, Pusan National University, Yangsan 50612, Republic of Korea; ^4^Institute of Medical Science, Ajou University School of Medicine, Suwon 16499, Republic of Korea

## Abstract

Yes-associated protein (YAP)/WW domain-containing transcription factor (TAZ) is critical for cell proliferation, survival, and self-renewal. It has been shown to play a crucial oncogenic role in many different types of tumors. In this study, we investigated the antitumor effect of the extracts of *Perilla frutescens* var. *acuta* (Odash.) Kudo leaves (PLE) on Hippo-YAP/TAZ signaling. PLE induced the phosphorylation of YAP/TAZ, thereby inhibiting their activity. In addition, the treatment suppresses YAP/TAZ transcriptional activity via the dissociation of the YAP/TAZ-TEAD complex. To elucidate the molecular mechanism of PLE in the regulation of YAP activity, we treated WT and cell lines with gene knockout (KO) for Hippo pathway components with PLE. The inhibitory effects of PLE on YAP-TEAD target genes were significantly attenuated in LATS1/2 KO cells. Moreover, we found the antitumor effect of PLE on MDA-MB-231 and BT549, both of which are triple-negative breast cancer (TNBC) cell lines. PLE reduced the viability of TNBC cells in a dose-dependent manner and induced cell apoptosis. Further, PLE inhibited the migration ability in MDA-MB-231 cells. This ability was weakened in YAP and TEAD-activated clones suggesting that the inhibition of migration by PLE is mainly achieved by regulating YAP activity. Taken together, the results of this study indicate that PLE suppressed cell growth and increased the apoptosis of breast cancer (BC) cells via inactivation of YAP activity in a LATS1/2-dependent manner.

## 1. Introduction

*Perilla frutescens* is a member of the Liliaceae/Labiatae (mint) family of flowering plants. Its leaves are consumed as a popular functional food in East Asian countries [1]. It has also been widely used as a traditional medicinal herb for expelling pathogens from the superficies and relieving exterior syndrome. The plant is used in traditional Chinese medicine (TCM) for pain due to blood stagnation caused by cold congealing. It has been used for various disorders such as depression-related diseases, asthma, anxiety, and tumors [1, 2].

Studies on *P. frutescens* have demonstrated its antioxidant [3], anti-inflammatory [4, 5], and anti-allergic properties [5, 6]. Several studies have reported that *P. frutescens* inhibits tumor growth and invasion in metastatic cancer cell lines and animal models [7–9]. Luteolin and triterpene acids, constituents of *P. frutescens,* have anti-inflammatory and antitumor effects [10, 11]. Although *P. frutescens* has been suggested to be a useful medicinal herb with antitumor activity, its exact molecular mechanisms are not yet fully understood.

The Hippo signaling pathway was first discovered in *Drosophila*, where it functions as a regulator of tissue and organ size [12]. Components of the Hippo pathway are highly conserved from *Drosophila* to humans, and a few mutations have been associated with human cancers [13]. The mammalian Hippo kinase cascade includes the mammalian STE20-like1/2 (MST1/2) and large tumor suppressor kinase 1/2 (LATS1/2) as well as the downstream effector Yes-associated protein (YAP) and WW domain-containing transcription factor (TAZ) [14]. YAP and TAZ are transcription co-activators without any DNA-binding domains; they enhance transcription by binding to specific transcription factors and in turn induce the expression of genes regulating cell proliferation, apoptosis, and differentiation [15]. The Hippo pathway inhibits both YAP and TAZ in a phosphorylation-dependent manner [16]. Abnormal YAP/TAZ overexpression and dysregulation of core Hippo kinases have been frequently observed in various human cancers [13, 17].

Triple-negative breast cancers (TNBCs) are an aggressive type of breast cancer (BC) that do not express receptors against estrogen or progesterone and HER2 [18, 19]. These tumors have a poorer prognosis than other types of BC due to the resistance to hormonal therapies or HER2 targeted therapies such as trastuzumab. Thus, intense studies are being carried out seeking the underlying mechanisms and new treatment approaches for TNBCs [20]. Recent studies reported that the Hippo pathway is highly activated in TNBCs and could be a driving force for the progression of TNBCs [21–24].

Numerous proteins have been implicated to act upstream of and regulate the Hippo pathway [14, 25]. However, the medicinal herbs that regulate the Hippo pathway are mostly unknown. Therefore, the aim of this study was to investigate the antitumor effects of *P. frutescens* on TNBC cells and its role in the Hippo-YAP signaling pathway.

## 2. Materials and Methods

### 2.1. Reagents and Antibodies

Anti-YAP (#14074), YAP/TAZ (#8414), phospho-YAP (Ser127, #4911), phospho-YAP (Ser397, #13619), LATS1 (#3477), LATS2 (#5888), phospho-LATS1/2 (Ser909/Ser872, #9157), phosphor-MST1/2 (Thr183/Thr180, #3681), MST2 (#3952), PARP (#9542), Caspase-3 (#14220), and pan-TEAD (#13295) were purchased from Cell Signaling Technology (Beverly, MA, USA). Anti-MST1 (No. 611052) was obtained from BD Transduction Laboratories (Lexington, KY, USA). Anti-Vinculin (V9131) was obtained from Sigma-Aldrich (St. Louis, MO, USA). Normal rabbit IgG for control immunoprecipitation (sc-2027) and anti-HA (sc-7392) were purchased from Santa Cruz Biotechnology, Inc. (Santa Cruz, CA, USA). Horseradish peroxidase-conjugated secondary antibodies (NA931V and NA934V) were purchased from GE Healthcare (Chicago, IL, USA). Phos-tag-conjugated polyacrylamide was purchased from Wako Pure Chemical Industries, Ltd. (Osaka, Japan). All chemicals and solvents used were purchased from Merck (Kenilworth, NJ, USA) and Sigma-Aldrich, unless stated otherwise. Polyvinylidene fluoride (PVDF) membrane (Immobilon-P) was obtained from Millipore Co. (Billerica, MA, USA). SuperSignal^®^ West Pico Chemiluminescent Substrate was obtained from Thermo Fisher Scientific, Inc. (Waltham, MA, USA).

### 2.2. Cell Culture and Transfection

HEK293T, LATS1/2 KO HEK293A, MST1/2 KO HEK293A, MCF7, MDA-MB-468, Hs578T, and SUM159 cells were cultured in Dulbecco's modified Eagle's medium (DMEM) containing 10% of fetal bovine serum (FBS), 50 units/mL of penicillin, and 50 *µ*g/mL of streptomycin (Gibco, MA, USA). MDA-MB-231, BT549, T47D, and SKBR3 cells were cultured in RPMI1640 containing 10% FBS, 50 units/mL penicillin, and 50 *µ*g/mL streptomycin. MCF10A cells were cultured in DMEM/F12 containing 5% horse serum (HS), 20 ng/mL of EGF, 0.5 *µ*g/mL of hydrocortisone, 10 *µ*g/mL of insulin, 100 ng/mL of cholera toxin, and 50 units/mL of penicillin, and 50 *µ*g/mL of streptomycin. MDA-MB-436 cells were cultured in DMEM/F12 containing 10% of FBS, 50 units/mL of penicillin, and 50 *µ*g/mL of streptomycin. HEK293A and MAP4K4/6/7 KO cells, kindly provided by Dr. Kun-Liang Guan's laboratory at the University of California San Diego, were maintained in DMEM with 10% FBS [26]. The cells were grown at 37°C in a humidified incubator in an atmosphere of 5% CO_2_. The human BC cell lines were purchased from ATCC (Manassas, VA) and Korean Cell Line Bank (KCLB; Seoul, South Korea). For plasmid DNA transfection, the cells were seeded on plates and incubated overnight. The cells were then transfected with a suitable concentration of each plasmid DNA using polyethyleneimine (PEI; Polysciences, Inc., Warrington, PA, USA) according to the manufacturer's instructions.

### 2.3. Retrovirus Infection and Stable Cell Lines

MDA-MB-231 cells were infected with retrovirus with 5 *µ*g/mL polybrene (Millipore Co.). Retroviral infection was performed as previously described [27]. Infected cells were selected with 2 mg/mL G418 (Gold Biotechnology, Inc., St Louis, MI, USA).

### 2.4. Generation of Knockout Cells

pSpCas9 (BB)-2A-Puro (PX459; plasmid #62988) and lenti CRISPR v2 vector (plasmid #52961) were supplied by Addgene (Watertown, MA, USA). The nucleotide guide sequences were designed using the CRISPR design tool at http://www.rgenome.net/cas-designer/. Single-guide RNAs (sgRNAs) were cloned into the PX459 and Lenti CRISPR V2 expression vector. HEK293A cells were transfected with cloned PX459. BT549 cells were infected with lentivirus generated from Lenti CRISPR V2 vector. Transfection was performed according to the manufacturer's instructions. After 24 h of transfection or infection, HEK293A and BT549 cells were selected for 2 days with media containing puromycin (InvivoGen, San Diego, CA, USA). The cells were then incubated with new media for 24 h before single cell sorting with BD FACS Aria™ III sorter (Franklin Lakes, NJ, USA) at the Three-Dimensional Immune System Imaging Core Facility.

Guide sequences were as follows:

  LATS1, 5′-GCAGCCATCTGCTCTCGTCG-3′
  LATS2, 5′-GTAGGACGCAAACGAATCGC-3′
  MST1, 5′-TCCTGGAGGTCTGATTCCAC-3′
  MST2, 5′-TGCTCCGTTCCTAAGGCAGA-3′

### 2.5. Preparation of *P. frutescens* Extract

*P. frutescens* leaf extract (PLE) was provided by the National Institute of Horticulture Herbal Science (NIHHS; Wanju, South Korea) of this institute. PLE powder was dissolved in absolute ethanol at a concentration of 10 mg/mL and used after filtration with a 0.45 *µ*m syringe filter.

### 2.6. High-Performance Liquid Chromatography (HPLC) Assay

The concentration of polyphenolic substances in PLE was determined by HPLC. The analyses were performed using Agilent 1260 Infinity II with a ZORBAX Eclipse Plus C18 (4.6 × 250 mm, 5 *µ*m, 95 Å; Agilent Technologies, Inc., Santa Clara, CA, USA). The mobile phase contained 0.1% phosphoric acid (solvent A) and acetonitrile (solvent B). The flow rate was 0.8 mL/min, and the injection volume was fixed at 10 *μ*l. The column temperature was maintained at 35°C during the separation, and the optical density of the effluent was monitored at 330 nm. Commercial *β*-carotene, caffeic acid, luteolin, quercetin, rosmarinic acid, and linolenic acid were purchased from Sigma-Aldrich and used as standards.

### 2.7. Western Blot and Immunoprecipitation Assay

Western blotting was performed using a standard protocol [27]. For immunoprecipitation, cells were lysed in a mild lysis buffer (10 mM Tris at pH 7.5, 100 mM NaCl, 1 mM EDTA, 1% NP-40, 50 mM NaF, 1 mM Na_3_VO_4_, and protease inhibitor cocktail [Sigma-Aldrich]) and centrifuged at 4°C at 12,000 × g. The supernatants were immunoprecipitated with appropriate antibodies for 2 h at 4°C, followed by the addition of Protein A/G magnetic beads (Thermo Fisher Scientific, Inc.) for an additional 1 h at 4°C. The immunoprecipitates were washed three times with the mild lysis buffer, and then the samples were eluted with SDS-PAGE sample buffer.

### 2.8. Luciferase Assay

HEK293A cells were seeded on 12-well plates, and after 12 h, they were transfected with 5×UAS-Luc reporter, pRL reporter, Gal4-TEAD4, and Flag-YAP2. On the next day, the cells were treated with PLE for 10 h and lysed. Luciferase activity was measured using the Dual-Glo® luciferase reporter assay kit (Promega, Madison, WI, USA) according to the manufacturer's instructions. The relative firefly luciferase activity was normalized to that of *Renilla* luciferase.

### 2.9. RNA Isolation and Real-Time Quantitative Polymerase Chain Reaction (RT-qPCR)

RNA isolation was performed using an RNeasy mini kit (QIAGEN, Germantown, MD, USA). Equal amounts of cDNA were synthesized from 1 *µ*g of total RNA using reverse transcriptase (Promega) with random hexamers (Takara Bio Inc., Shiga, Japan) and necessary reagents for synthesis. qPCR was performed using the SYBR Green PCR master mix (Sigma-Aldrich) with an appropriate primer pair, following the manufacturer's protocol. The normalization of several YAP-dependent genes was done using hypoxanthine-guanine phosphoribosyltransferase 1 gene (HPRT). The primers used were as follows:

  HPRT, 5′-AGAATGTCTTGATTGTGGAAGA/ACCTTGACCATCTTTGGATTA-3′
  CTGF, 5′-CCAATGACAACGCCTCCTG/TGGTGCAGCCAGAAAGCTC-3′
  CYR61, 5′-AGCCTCGCATCCTATACAACC/TTCTTTCACAAGGCGGCACTC-3′
  INHIBA, 5′-TTGCCAACCTCAAATCGTGCT/CCCACACTCCTCCACGATCAT-3′
  CXCL1, 5′-CCACTGAACTGCGCTGCCA/AGCTTTCCGCCCATTCTTGAG-3′
  FSTL1, 5′-GCAGCAACTACAGTGAAATCC/ATGGCAGTTTCATTCTGTTCC-3′
  PAPPA, 5′-TCTTGGTCACTGATGGTGTGA/AGAGGTCTCCGGCAGTGAT-3′

### 2.10. Wound Healing Assay

For the wound healing assay, the cells were plated in 6-well plates and allowed to reach approximately 80% confluence. Wounds were then generated by scratching the surface using a Scar™ scratcher (SPL Life Sciences Co., Ltd., South Korea), followed by washing with phosphate-buffered saline (PBS) to remove the cell debris. The cell medium was replenished with or without PLE and allowed to migrate to the wound area for 36 h. The wound regions were photographed every 12 h, and the images were captured under a phase-contrast microscope (OLYMPUS 1X71 DP controller, Tokyo Japan). The average wound widths at 0 and 36 h time points were quantified using the ImageJ program (National Institutes of Health, Bethesda, MD, USA).

### 2.11. Cytotoxicity Assay

Cell viability was assessed using the EZ-Cytox cell viability assay kit (Daeil Lab Service, Seoul, South Korea). Briefly, HEK293A, MCF10A, MDA-MB-231, and BT549 cells were added to each well of a 96-well plate. After incubation for 24 h, the cells were treated with PLE for 24 h. Then, 10 *μ*L EZ-Cytox solution was added to each well of the plate and the cells were incubated at 37°C for 2 h. Subsequently, the absorbance was measured at 450 nm using a microplate spectrophotometer (BioTek Instruments, Winooski, VT, USA). Cell viability (%) was calculated using the formula [*A*_*s*_/*A*_*c*_] × 100, where *A*_*s*_ is the absorbance of the well-containing cells, culture medium, EZ-Cytox solution, and stimulants and *A*_*c*_ is the absorbance of the well-containing cells, culture medium, and EZ-Cytox solution.

### 2.12. Sulforhodamine B (SRB) Assay

HEK293A, MCF10A, MDA-MB-231, and BT549 cells were seeded into each well of a 96-well plate. After incubation for 24 h, the cells were treated with DMSO and 100 *μ*g/mL of PLE for up to 5 days; the cell medium with serially diluted PLE was refreshed every 2 days. The plates were harvested every 2 days at the same time and fixed with 10% trichloroacetic acid (TCA) solution (Sigma-Aldrich) at 4°C overnight. The plates were washed with distilled water more than three times. After that, the plates were stained with 0.4% SRB (Sigma-Aldrich) in 1% acetic acid solution for 30 min at 25°C. The cells were then washed with 0.1% acetic acid solution and dried at 25°C for 1 h. Finally, 10 mM Trizma base (Sigma-Aldrich) was added, and the plates were incubated on a rocker for 1 h to resolve the stained cells. The SRB levels were measured using a microplate spectrophotometer (BioTek Instruments) at an absorbance of 540 nm.

### 2.13. Clonogenic Growth Assay

MDA-MB-231 cells and TEAD1ΔC-YAP (AD)-expressing cells were seeded in a 6-well plate. After 24 h, the cells were treated with 10 and 25 *μ*g/mL of PLE for 10 days. Then, the cells were fixed with 4% formaldehyde for 10 min and stained with 0.25% crystal violet for 12 h. After washing three times with distilled water, the cells were destained with 95% ethanol for 1 h. The absorbance of the 100 *μ*L destaining solution was measured at 595 nm using a microplate spectrophotometer (BioTek Instruments).

### 2.14. Statistical Analysis

Values are expressed as the mean ± standard error of the mean (SEM) of three samples obtained from three independent experiments. Student's *t*-test (unpaired, one-tailed) was performed to calculate a *p*-value using Prism 5.0 software (GraphPad Software, La Jolla, CA, USA) and considered at ^*∗*^*p* < 0.05, ^*∗∗*^*p* < 0.01, and ^*∗∗∗*^*p* < 0.001.

## 3. Results

### 3.1. Analysis of PLE using HPLC

In this study, we screened medicinal herb extracts to identify novel inhibitors of YAP/TAZ. Through a review of the literature, 13 medicinal herbs were selected to screen for possible inhibitory activity against YAP/TAZ through the Hippo tumor suppressor pathway. To determine the effects of the medicinal herb on YAP activation, we evaluated the YAP phosphorylation status using mobility shift on a phos-tag gel. A pilot screening of the medicinal plant extracts identified that PLE potently inhibited YAP by promoting its phosphorylation (Figure S1).

**Figure 1:**
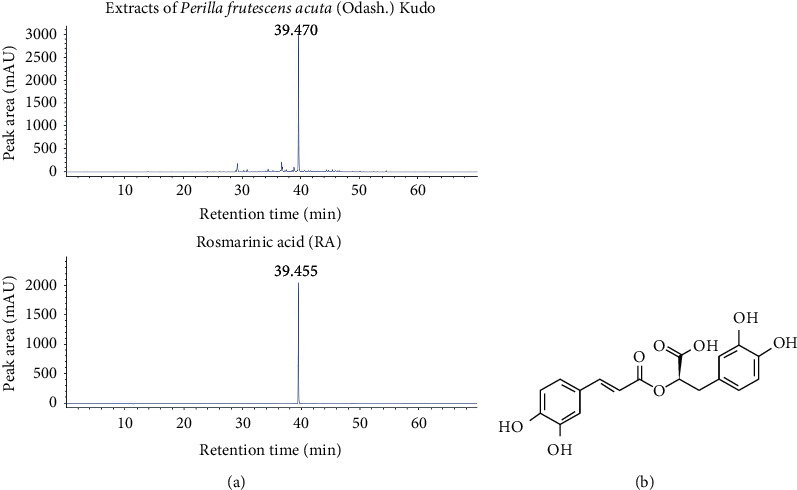
Flavonoid profiles of the extracts of *Perilla frutescens* var. *acuta* (Odash.) Kudo leaves (PLE). (a) Comparative high-performance liquid chromatography ultraviolet (HPLC-UV) chromatograms of PLE ethanol extracts (top) and rosmarinic acid (RA, bottom) in the standard solution were obtained using an Agilent 1260 series liquid chromatography system. Chromatographic separation was carried out on a ZORBAX Eclipse Plus C18 column (4.6 × 150 mm, 5 *μ*m), and the column temperature was maintained at 35°C. The mobile phase consisted of water containing 0.1% phosphoric acid (A) and acetonitrile (B). The composition of the mobile phase was 0–4 min, 0% (B); 10 min, 4% (B); 20 min, 10% (B); 30 min, 20% (B); 40 min, 40% (B); and 70 min, 100% (B). RA was detected at concentrations ranging from 38 to 40 min. PLE contained 4.6 mg/g of RA. (b) The chemical structure of RA isolated from PLE.

Phytochemical constituents such as catechin, ferulic acid, apigenin, luteolin, rosmarinic acid, and caffeic acid have been identified from *P. frutescens* [28]. Therefore, in this study, commercially available *β*-carotene, caffeic acid, luteolin, quercetin, rosmarinic acid, and linolenic acid were used as standard compounds for HPLC-UV analysis. The quantitative profiles of the PLE composition are shown in Figure 1(a). Rosmarinic acid was exclusively detected in the range of 38–40 min and at 4.6 mg per gram of PLE (Figure 1(b) and Table 1).

### 3.2. PLE Increases YAP Phosphorylation and Reduces YAP-TEAD-Mediated Transcriptional Activity

**Figure 2:**
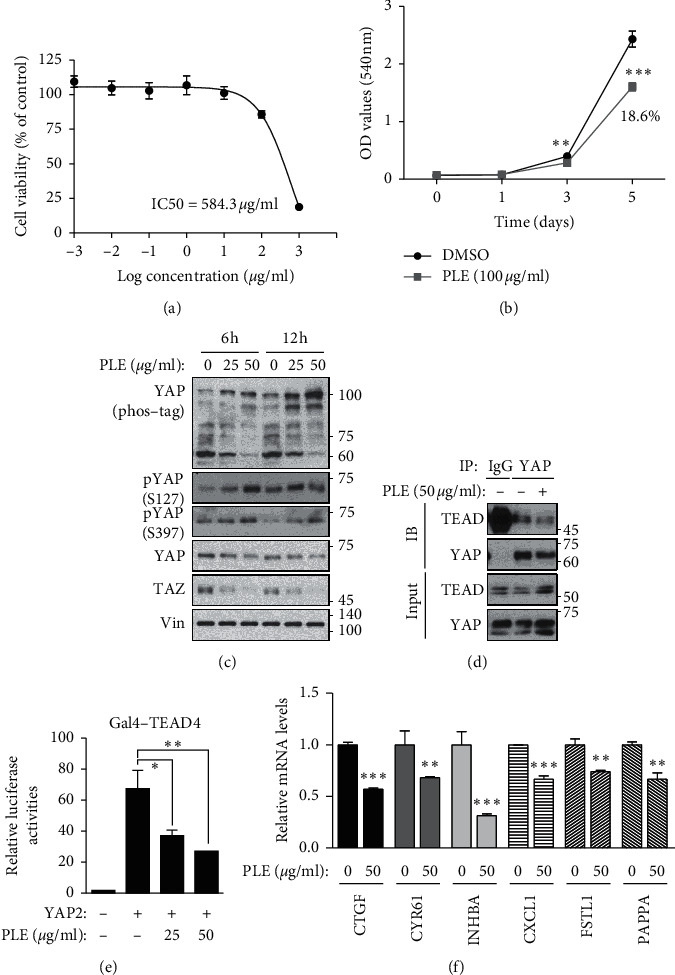
PLE inhibits YAP activity and disrupts YAP-TEAD complex to inhibit its transcriptional activity. HEK293A cells were treated using the indicated concentrations of PLE for 24 h. (a) Cell viability was measured using an EZ-Cytox cell viability assay kit. IC50 values were calculated from concentration response curves using Prism 5.0 software. The IC50 value of HEK293A cells was 584.3 *μ*g/mL. Error bars represent ± SEM from *n* = 3 per group. (b) HEK293A cells were treated with DMSO and 100 *μ*g/mL of PLE for 5 days, and cell proliferation was measured by SRB assay at an absorbance of 540 nm. The proliferation of HEK293A cells with PLE was decreased by 18.6% compared with control cells. Error bars represent mean ± SEM (*n* = 3). ^*∗∗∗*^*p* < 0.001, ^*∗∗*^*p* < 0.01; Student's *t*-test (unpaired, one-tailed) was used for statistical analysis. (c) HEK293A cells were treated with various concentrations (50 and 100 *μ*g/mL) of PLE for 2 and 6 h, YAP phosphorylation states were determined by phos-tag gel shift assay, and cell lysates were subjected to immunoblotting with anti-YAP, pYAP, TAZ, and Vin. (d) HEK293A cells were treated with PLE (50 *μ*g/mL) for 6 h. Endogenous YAP/TAZ was immunoprecipitated, and the co-precipitated pan-TEAD was detected by western blot. (e) HEK293A cells were co-transfected with 5 × UAS-luciferase reporter, Gal4-TEAD4, Renilla, with or without HA-YAP2. HEK293A cells were treated with PLE in a concentration-dependent manner. Cells were harvested 10 h after treatment. Representative results of a single experiment with *n* = 3 biological replicates; three independent experiments were carried out. (f) HEK293A cells were treated with 50 *μ*g/mL of PLE for 12 h before harvesting. mRNA levels of CTGF, CYR61, INHBA, CXCL1, FSTL1, and PAPPA were determined using RT-qPCR. Bars represent mean ± SEM (*N* = 3). ^*∗*^*p* < 0.05, ^*∗∗*^*p* < 0.01, and ^*∗∗∗*^*p* < 0.001; Student's *t*-test (unpaired, one-tailed) was used for statistical analysis.

To investigate the molecular mechanisms associated with the Hippo-YAP pathway, we treated HEK293A cells with the PLE. The IC50 value of HEK293A was 584.3 *μ*g/mL (Figure 2(a)), and the percentages of cell proliferation were 18.6% lower when treated with 100 *μ*g/mL of PLE, compared to those with control (Figure 2(b)). Expression of pYAP, YAP, and TAZ in PLE-treated HEK293A cells was determined by immunoblotting. As shown in Figure 2(c), HEK293A cells showed increased phosphorylation of YAP at Ser127 and Ser397 following PLE treatment. Activated LATS1/2-driven phosphorylation on both Ser127 and Ser397 is an important step for nuclear/cytosol translocation and protein degradation [16, 29]. PLE significantly reduced YAP and TAZ levels in HEK293A cells in a concentration-dependent manner. These results are consistent with those of previous studies showing that phosphorylated YAP and TAZ are sequestered and degraded in the cytoplasm, thus revealing that PLE could suppress the function of YAP by upregulating the level of phosphorylation [30].

Many transcription factors, including TEA domain family members (TEADs), RUNX2, TBX5, Oct4, and ErbB4, have been implicated as YAP partners [31, 32]. In mammalian cells, the TEADs (TEAD1–4) are major partners mediating YAP/TAZ biological functions [33]. When Hippo signaling is not activated, YAP and TAZ are not phosphorylated. These unphosphorylated YAP and TAZ enter the nucleus and bind to the transcription factor TEAD to induce gene transcription [34]. To determine the effects of PLE on YAP/TAZ and TEAD binding, we conducted a co-immunoprecipitation assay in HEK293A cells. Consistent with YAP phosphorylation, PLE blocked the interaction between YAP and TEAD (Figure 2(d)). To ascertain the effects of PLE on the YAP-TEAD transcriptional complex, we evaluated its transcriptional activity using the luciferase reporter system (Figure 2(e)). PLE also effectively blocked the ability of YAP to activate TEAD-mediated transcription but did not abolish its ability to activate RUNX2 (Figure S2).

To further confirm the negative regulation of YAP by PLE, we measured YAP-TEAD-driven target gene expression. We found that PLE significantly diminished the expression of YAP-TEAD target genes, including CTGF, CYR61, INHBA, CXCL1, FSTL1, and PAPPA, in a dose-dependent manner (Figure 2(f)). Collectively, our data suggest that PLE inhibited YAP activity via disruption of the YAP-TEAD transcriptional complex.

### 3.3. PLE Inhibits YAP/TEAD Transcriptional Activity via LATS1/2-Dependent Mechanisms but Not MST1/2-Dependent Mechanisms

**Figure 3:**
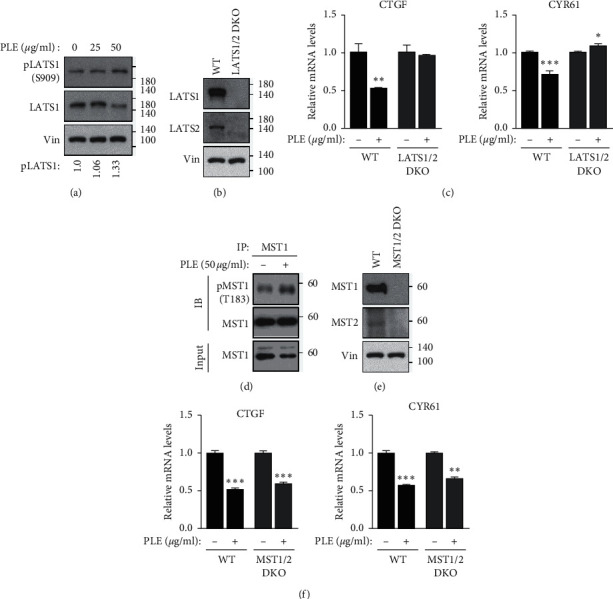
PLE suppressed YAP target genes in a LATS1/2-dependent manner. (a) HEK293A cells were treated with increasing concentrations of PLE for 2 h. Cell lysates were immunoblotted using anti-LATS1 and anti-pLATS1 (S909) antibodies. Quantifications of western blots for pLATS1 (S909) were performed by ImageJ program. (b, c) Wild-type and LATS1/2 knockout (KO) HEK293A cells were treated with PLE. After 12 h, mRNA levels of CTGF and CYR61 were measured using RT-qPCR. Error bars represent ± SEM from *n* = 3 per group. (d) HEK293A cells were treated with increasing concentrations of PLE for 2 h. Cell lysates were immunoblotted using anti-MSTS1 and anti-pMST1 (T183) antibodies. (e, f) Wild-type and MST1/2 KO HEK293A cells were treated with PLE for 12 h and mRNA levels of CTGF and CYR61 were measured using RT-qPCR (error bars represent ± SEM from *n* = 3 per group). Cell lines were immunoblotted to analyze indicated antibodies. ^*∗*^*p* < 0.05, ^*∗∗*^*p* < 0.01, and ^*∗∗∗*^*p* < 0.001; Student's *t*-test (unpaired, one-tailed) was used for statistical analysis In Figure 3(a)), the numbers of size maker depart from indicated lines.

Among the Hippo signaling core kinases that regulate YAP/TAZ activity, LATS1/2 is one of the most significant kinases for YAP. Phosphorylation of Ser909 and Thr1079 is essential for LATS1 kinase activity [35]. Following PLE treatment, the increase in YAP phosphorylation could be due to LATS1 kinase activity. To test this hypothesis, we monitored Ser909 phosphorylation in endogenous LATS1 in response to PLE. Phosphorylation of Ser909 in LATS1 was increased by PLE treatment (Figure 3(a)). Next, we attempted to determine whether PLE downregulates the transcriptional activity of YAP/TAZ via LATS1/2 using the LATS1/2^−/−^ (LATS1/2 KO) HEK293A cells generated by the CRISPR-Cas9 system Figure 3(b). The transcriptional activation of YAP target genes such as CTGF and CYR61 was suppressed by PLE treatment. However, no noticeable changes in the expression levels of YAP target genes were observed between PLE-treated and untreated groups in LATS1/2 KO cells (Figure 3(c)). This suggests that PLE regulates target gene expression via LATS1/2-dependent mechanisms.

MST1/2 is autophosphorylated on multiple sites in response to various stress stimuli, which results in the activation of MST1/2 [36]. Among these multiple autophosphorylation sites, phosphorylation at Thr183 and Thr180 is essential for Mst1 and Mst2 activation, respectively [37]. Following PLE treatment, the increase in LATS1/2 phosphorylation could be due to MST1/2 activation. To test this hypothesis, we monitored Thr183 phosphorylation. The phosphorylation of Thr183 in MST1 was slightly increased by PLE treatment (Figure 3(d)). Next, to confirm the role of MST1/2 in PLE-induced YAP regulation, we compared YAP target gene expression between wild-type and MST1/2^−/−^ (MST1/2 KO) HEK293A cells (Figure 3(e)). As expected, PLE treatment decreased the expression of CTGF and CYR61 (Figure 3(f)). However, PLE still reduced the expression of CTGF and CYR61 in MST1/2 KO cells, suggesting that PLE reduces YAP target gene levels independent of MST1/2.

Although MST1/2 is well characterized as an upstream activating kinase of LATS1/2 [35], accumulating evidence indicates that MST1/2 and MAP4Ks directly phosphorylate and activate LATS1/2 in a partially redundant manner [26, 38, 39]. Next, to confirm the role of MAP4Ks in PLE-induced YAP regulation, we compared YAP target gene expression between wild-type and MAP4K4/6/7^−/−^ (MAP4K4/6/7 KO) HEK293A cells. PLE treatment decreased the expression levels of CTGF and CYR61, but MAP4K4/6/7 deletion did not alter the expression levels of these genes in response to PLE treatment, suggesting that PLE could reduce YAP target gene levels dependent on MAP4K4/6/7 (Figure S3). In this study, we discovered a novel LATS1/2-dependent mechanism for the inhibitory effect of PLE on YAP/TAZ phosphorylation and inactivation, but MAP4Ks had a redundant function in activating LATS1/2 kinases.

### 3.4. Induction of Cytotoxicity and Apoptosis by PLE in TNBC Cells MDA-MB-231 and BT549

The Hippo-YAP signaling pathway has attracted much attention in cancer research since the Hippo-YAP signaling pathway, a well-known regulator of organ size, has been known to play an essential role in cancer development [40, 41]. The regulation of Hippo-YAP represents a promising strategy for the treatment of various cancers [21, 42]. Therefore, we further explored the effects of PLE on Hippo-YAP signaling in TNBC cells. Western blotting was performed to determine the levels of YAP and TAZ protein in 10 BC cell lines. YAP and TAZ were overexpressed in the MDA-MB-231, BT549, and MDA-MB-468 cell lines, whereas their expression levels were relatively low in the MCF7 and MDA-MB-436 cell lines. MDA-MB-231 and BT549 have been reported to have high invasion potential; therefore, these two cell lines were selected for further study (Figure S4).

**Figure 4:**
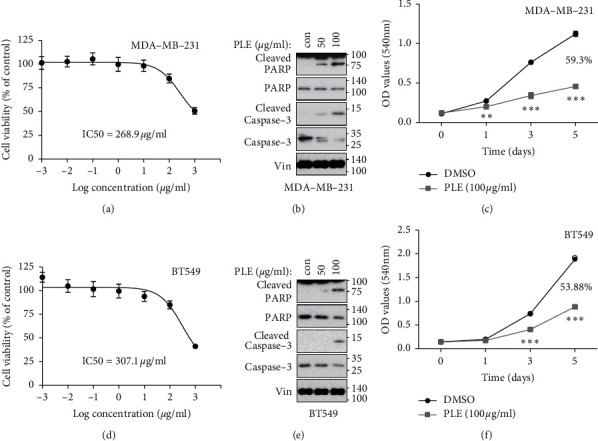
Cytotoxic, apoptosis-inducing, and antiproliferative effects of PLE on MDA-MB-231 and BT549. (a, d) MDA-MB-231 and BT549 cells were treated with various concentrations (0.001, 0.01, 0.1, 1, 10, 100, and 1000 *μ*g/mL) of PLE for 24 h. Cell viability was measured by using the EZ-Cytox cell viability assay kit. IC50 values were determined from concentration response curves using Prism 5.0 Software. The IC50 value of MDA-MB-231 and BT549 cells was 268.9 *μ*g/ml and 307.1 *μ*g/ml, respectively. Error bars represent ± SEM from *n* = 3 per group. (b, e) To measure the levels of apoptosis-related protein, western blot was performed on MDA-MB-231 and BT549 cells treated with the PLE at 50 and 100 *μ*g/mL for 12 h. The lysates were immunoblotted with anti-PARP, cleaved PARP, Caspase-3, and cleaved Caspase-3. (c, f) MDA-MB-231 and BT549 cells were treated with DMSO and 100 *μ*g/mL PLE, and the medium with diluted PLE was refreshed every two days. After treatment for another 5 days, cell proliferation was determined by using SRB assay at an absorbance of 540 nm. The proliferation of MDA-MB-231 and BT549 cells with PLE was decreased by 59.3% and 53.88%, respectively, compared with control cells. Bars represent mean ± SEM (*n* = 3). ^*∗∗∗*^*p* < 0.001, ^*∗∗*^*p* < 0.01; Student's *t*-test (unpaired, one-tailed) was used for statistical analysis.

To investigate whether PLE has a better cytotoxic effect on TNBC compared to normal human mammary epithelial cell line, the cytotoxic activity of PLE was examined using MTT assay in MDA-MB-231, BT549, and MCF10A cells with various concentrations of PLE for 24 h. The results showed that PLE significantly inhibited the growth of MDA-MB-231 and BT549 cells to a greater extent compared with that of MCF10A cells in a concentration-dependent manner, with IC50 values of 268.9 *μ*g/mL (Figure 4(a)), 307.1 *μ*g/mL (Figure 4(d)), and 680.5 *μ*g/mL (Figure S5(a)), respectively. The IC50 values showed that MCF10A cells were more resistant to the cytotoxic effect of PLE than both TNBC cells. These results showed that PLE has a stronger cytotoxic effect on TNBC cell lines than on non-malignant MCF10A cells. We then examined the effect of PLE on cell apoptosis. Because the cleavage of several key proteins, such as Caspase-3 and PARP, is considered to be a hallmark of apoptosis, we measured the levels of cleaved Caspase-3 and PARP in MDA-MB-231 and BT549 cells at PLE concentrations of 50 and 100 *μ*g/mL. Western blot analysis revealed significant upregulation of cleaved Caspase-3 and PARP in both BC cell lines in a dose-dependent manner (Figures 4(b) and 4(e)).

Since PLE decreased TNBC cell viability and increased cell apoptosis effectively, we next investigated the suppressive effect of PLE on TNBC cell proliferation. MCF10A, MDA-MB-231, and BT549 cells were treated with 100 *μ*g/mL PLE for 5 days, and their proliferation ability was assessed using the SRB assay. As shown in Figures 4(c) and 4(f), PLE markedly inhibited the proliferation of MDA-MB-231 and BT549 cells to a greater extent compared with that of MCF10A cells (Figure S5(b)). Collectively, these results indicate that PLE significantly inhibited the growth of MDA-MB-231 and BT549 cells.

### 3.5. PLE Blocks Tumor Progression of Human BC Cells by Repressing YAP Activity

**Figure 5:**
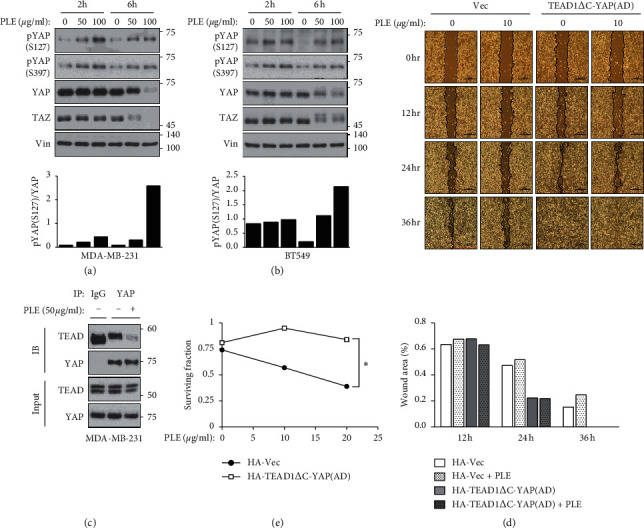
Constitutively active YAP-TEAD rescued PLE-induced inhibition of cancer cell migration and proliferation. (a, b) To determine the effect of PLE on the YAP phosphorylation, MDA-MB-231 and BT549 cells were treated with PLE (25 and 50 *μ*g/mL) for 6 and 12 h. Cell lysates were subjected to immunoblotting with the indicated antibodies. The bar graphs show the pYAP (S127) amounts normalized to total YAP amounts for individual samples. (c) HEK293A cells were treated with PLE (50 *μ*g/mL) for 6 h. Endogenous YAP/TAZ was immunoprecipitated, and the co-precipitated pan-TEAD was detected by western blot. (d) Mobility of MDA-MB-231 cells expressing the HA-vector or TEAD1ΔC-YAP (AD) was evaluated using the wound healing assay. The cells were wounded and treated with PLE (10 *μ*g/mL) for 36 h in a serum-containing medium. At different time points (0, 12, 24, and 36 h), phase-contrast photographs of the wounds were taken and analyzed using ImageJ program. (e) MDA-MB-231 cells expressed with HA-vector or TEAD1ΔC-YAP (AD) were treated with PLE at concentrations of 10 and 25 *μ*g/mL. The cells were applied to clonogenic growth assay after 10 days of the treatment. In the clonogenic growth assay, DMSO-treated cells served as a control. Each value is expressed as mean ± SEM from *n* = 3 per group. ^*∗*^*p* < 0.05, ^*∗∗*^*p* < 0.01; Student's *t*-test (unpaired, one-tailed) was used for statistical analysis. In Figure 5(b), the numbers of size maker depart from indicated lines.

We further explored the effects of PLE on Hippo-YAP signaling in TNBC cells. We examined the levels of phosphorylated YAP in treated BT549 and MDA-MB-231 cells. As shown in Figures 5(a) and 5(b), PLE induced phosphorylation and degradation of YAP as well as TAZ in MDA-MB-231 and BT549 cells in a dose-dependent manner. At least one TEAD gene is expressed in most adult tissues [43–45], and all four TEADs are abundantly expressed in TNBC cells as indicated by the expression profiles of the TEAD extracted from the Cancer Cell Line Encyclopedia (CCLE) (Figure S6). To determine the effects of PLE on YAP/TAZ and TEAD binding, we conducted a co-immunoprecipitation assay in MDA-MB-231 cells. Consistent with YAP phosphorylation, PLE blocked the interaction between YAP and TEAD (Figure 5(c)), strengthening the notion of the link between PLE and YAP inhibition. YAP enhances several processes responsible for tumor growth and metastasis, such as cellular proliferation, migration, and invasion [46, 47]. Therefore, we performed a wound-healing assay to determine whether PLE inhibits the migration of MDA-MB-231 cells. As expected, PLE suppressed the migration of MDA-MB-231 cells (Figure 5(d)). To ascertain the effects of PLE on the YAP-TEAD transcriptional complex and thereby confirm whether the inhibition of migration by PLE was due to the inhibition of YAP-TEAD, we constructed MDA-MB-231 cells expressing an active YAP-TEAD fusion protein (TEAD1ΔC-YAP (AD)) (Figure S7). The migration inhibitory effect of PLE disappeared in TEAD1ΔC-YAP (AD)-expressing MDA-MB-231 cells. This result suggests that inhibition of migration by PLE is achieved by inhibiting YAP-TEAD activity (Figure 5(d)). Further, to evaluate the biological effects of PLE on the growth of MDA-MB-231 cells, we performed a clonogenic growth assay in both the vector and TEAD1ΔC-YAP(AD) expressing MDA-MB-231 cells. The ability of PLE to inhibit cell growth also disappeared in MDA-MB-231 cells expressing TEAD1ΔC-YAP (AD) (Figure 5(e)). Collectively, these results demonstrate that PLE suppressed tumor progression by modulating YAP activity.

These findings were further confirmed by examining the cytotoxic activity and the level of cleaved PARP1 in BT549 WT and LATS1/2 KO cells generated by the CRISPR-Cas9 system (Figure 3(b)). The MTT assay showed that BT549 LATS1/2 KO cells were more resistant to the cytotoxicity of PLE than BT549 WT cells after treatment with PLE at increasing concentrations for 24 h. The IC50 value of BT549 WT was 307.1 *μ*g/mL (Figure 4(d)), and the BT549 LATS1/2 KO showed an IC50 value of 713 *μ*g/mL (Figure S8(a)). Western blot results showed that PLE increased the levels of cleaved PARP1 proteins in BT549 cells to a greater extent compared with those in BT549 LATS1/2 KO cells (Figure S8(b)). These findings imply that PLE requires LATS1/2 to repress YAP/TAZ signaling.

## 4. Discussion

BC is the most frequently occurring cancer among women, accounting for 25% of all cancer cases worldwide. Despite the substantial efforts made in the past decades to improve survival and quality of life, BC remains a deadly threat for patients [48]. TNBCs, characterized by the absence of estrogen, progesterone, and ErbB2 receptors, account for approximately 10–15% of all BCs and are an aggressive type of BC. They have a poorer prognosis than other types of BCs. TNBC has an inadequate response to tamoxifen or trastuzumab, which has been developed to treat estrogen receptor-positive (ER+) BC or HER2 positive BC [49, 50]. In addition to the limitations of targeted therapies, the appearance of drug resistance to monotherapy is a major obstacle to the treatment of TNBC [18–20]. Thus, there is a continuing need to explore new therapeutic targets in BC. In addition, the discovery of therapeutic agents for these targets is still essential in chemotherapy for BC.

The role of YAP in organ size control or tumor development has been confirmed in mammals using transgenic mouse models [12, 51]. YAP and TAZ are cotranscription factors between the cytoplasm and nucleus. They are known to induce cell proliferation and antiapoptotic genes by binding to several transcription factors, particularly TEADs [41]. When Hippo, the core upstream kinase of YAP/TAZ, is activated, it phosphorylates LATS1/2, which in turn inactivates YAP/TAZ via phosphorylation. Conversely, when the Hippo kinase system is off, unphosphorylated YAP/TAZ enters the nucleus and expresses target genes associated with cell proliferation [34].

Activation of YAP/TAZ has been reported in various cancers [13, 52]. YAP/TAZ plays a particular role in the transformation of healthy epithelial cells into metastasized cancer cells through the EMT process [53]. In particular, YAP/TAZ activity is proportionally correlated with the grade and stage of BC [54], and nuclear expression of TAZ is strongly associated with TNBC [55]. YAP enhances tumor progression and metastasis in BC cells [47]. Thus, YAP/TAZ has an oncogenic role in the migration and invasion of BC cells [56]. Strategies to inhibit YAP/TAZ activity or activate Hippo signaling can be beneficial in treating various cancers, including TNBC.

Natural medicinal products are derived from living organisms, such as plants, animals, or microorganisms. Recently, natural compounds have gained importance with an improved understanding of their pharmacological or biological effects and contributions for medicinal use [57]. Clinical studies have revealed that natural compounds have considerable promise against many diseases [58]. Thus, natural compounds with physiological functions have been identified as targeted interventions that can regulate the activity of key molecules of signaling pathways. Among the numerous natural plant products, *P. frutescens* has been widely used as a source of herbal materials in TCM. PLE is a functional and medicinal herb that has been widely consumed in Asian countries. Previous studies have reported that PLE has anti-oxidative, anti-inflammatory, and antitumor effects [2–7]. Several studies have revealed the presence of anthocyanins, flavonoids, and phenolic acids in *P. frutescens* [3, 8–11]. In addition, recent studies suggest that various compounds in PLE have anticancer activities. Luteolin, a flavonoid isolated from *P. frutescens* leaves, has been reported to inhibit tumor proliferation and metastasis and induce apoptosis via various cellular signaling pathways [59–61]. Rosmarinic acid was suggested as an active ingredient mediating anticarcinogenic effects in a mouse skin papilloma model and exerting anti-inflammatory effects through its antioxidant activity [62–64].

Our results showed that PLE exerts an anticancer effect on MDA-MB-231 and BT549 cells. PLE not only inhibited the viability of TNBC cells (Figures 4(a) and 4(d)) but also reduced their proliferation and migration (Figures 4(c), 4(f), and 5(d)). Examining the effect of PLE on Hippo-YAP signaling revealed that PLE inhibited the activation of YAP/TAZ via phosphorylation (Figures 2(c), 5(a), and 5(b)). PLE also inhibited the binding between YAP and TEAD in HEK293A and MDA-MB-231 cells (Figures 2(d) and 5(c)). PLE downregulated the transcriptional activity of YAP-TEAD (Figures 3(c) and 3(f)). In particular, the inhibitory effects of PLE on the expression of YAP-dependent genes were also abolished in LATS1/2 KO cells (Figure 3(c)). The cytotoxicity and apoptosis-inducing ability of PLE were also significantly attenuated in the LATS1/2 knockout clone (Figures S8(a) and S8(b)). These results suggest that PLE exhibits anticancer effects by inhibiting the activity of YAP through LATS-dependent YAP phosphorylation.

In this study, the anticancer efficacy of PLE through inhibition of YAP activity was clarified using an in vitro model. Hence, further studies are needed to verify the efficacy of PLE in animal models and to develop new therapeutic strategies using PLE to regulate the growth and proliferation of TNBCs.

## Figures and Tables

**Table 1:** Retention time, calibration equation, and correlation coefficient for compound in extracts of *Perilla frutescens* var. *acuta* (Odash.) Kudo leaves.

Compound	Retention time (min)	Calibration equation	Correlation coefficient (*r*^2^)
Rosmarinic acid	39.455	*Y* = 57.769*x* + 215.250	0.999

^a^ *y*: peak area, *x*: concentration of standards (mg/mL).

## Data Availability

The data used in the study are available upon request to the corresponding author.

## Abbreviations

LATS1/2: Large tumor suppressor kinase 1/2
PLE: *Perilla frutescens* var. *acuta* (Odash.) Kudo leaf extracts
TAZ: WW domain-containing transcription factor
TNBC: Triple-negative breast cancer
YAP: Yes-associated protein.

## Supplementary Materials

Supplementary Materials Figure S1. *Perilla frutescens* var. *acuta* (Odash.) Kudo leaf extract (PLE) induces YAP phosphorylation. PLE increased YAP phosphorylation. HEK293A cells were treated with various natural compounds (50 *μ*g/mL) for 6 h. Dimethyl sulfoxide (DMSO) was used as a control treatment. Phosphorylated proteins were detected using a phos-tag gel. Vinculin was used as the loading control. Figure S2. PLE has no effect on YAP mediated RUNX2-luferase reporter activity. HEK293A cells were co-transfected with a 6 × OSE2-luciferase reporter for RUNX2, with or without HA-YAP2. HEK293A cells were treated with PLE in a concentration-dependent manner. Cells were harvested 10 h after treatment. Representative results of a single experiment with *n* = 3 biological replicates; three independent experiments were carried out. Luciferase activity was measured and normalized to that of co-transfected *Renilla*. Figure S3. PLE inhibited mRNA expression of YAP target genes in a MAP4K4/6/7-dependent manner. Wild-type and MAP4K4/6/7 KO HEK293A cells were treated with PLE for 12 h, and then mRNA levels of CTGF and CYR61 were measured using RT-qPCR (error bars represent ± SEM from *n* = 3 per group). *∗p* < 0.05, *∗∗p* < 0.01, and *∗∗∗p* < 0.001; Student's *t*-test (unpaired, one-tailed) was used for statistical analysis. Figure S4. Expression of YAP and TAZ on human BC and normal cell lines was analyzed by western blot. Cell lysates were subjected to immunoblotting with the indicated antibodies. Both YAP and TAZ were expressed predominantly in MDA-MB-231 and BT549 human BC cell lines. Figure S5. Effects of PLE on the viability of MCF10A cells. (a) MCF10A cells were treated using the indicated concentrations of PLE for 24 h. Cell viability was measured by using the EZ-Cytox cell viability assay kit. IC50 values were calculated from concentration response curves using Prism 5.0 software. The IC50 value of MCF10A cells was 680.5 *μ*g/mL. Error bars represent ± SEM from *n* = 3 per group. (b) MCF10A cells were treated with DMSO and 100 *μ*g/mL of PLE for 5 days. Cell proliferation was measured by SRB assay at an absorbance of 540 nm. MCF10A cells with PLE were decreased by 14% compared with that of the control cells. Bars represent mean ± SEM (*n* = 3). *∗∗∗p* < 0.001, *∗∗p* < 0.01; Student's *t*-test (unpaired, one-tailed) was used for statistical analysis. Figure S6. PLE disrupts YAP-TEAD interaction in MDA-MB-231 cells. (a) Levels of TEAD expression among several BC cell lines. Gene expression analysis of the Broad-Novartis Cancer Cell Line Encyclopedia (CCLE) dataset showed that TEAD1-4 were highly expressed in various BC cell types. The *x*-axis indicates cancer cell types; *y*-axis indicates the target expression levels of TEAD1–4 in BC cell lines. (b) MDA-MB-231 cells were treated with PLE (50 *μ*g/mL) for 6 h. Endogenous YAP/TAZ was immunoprecipitated, and the co-precipitated TEAD was detected by western blot. Figure S7. TEAD1ΔC-YAP (AD), a fusion of the TEAD DNA-binding domain with the YAP transactivation domain, was stably expressed in MDA-MB-231 cells. Expression of HA-pPGS (empty vector) and HA-TEAD1ΔC-YAP (AD) was confirmed using western blot, and the mRNA level of CYR61 was determined using RT-qPCR (error bars represent ± SEM from *n* = 3 per group). Figure S8. LATS1/2 KO BT549 cells increased resistance to PLE inhibitory effects to a greater extent compared with wild-type cells. (a) BT549 LATS1/2 KO cells were treated with various concentrations (0.001, 0.01, 0.1, 1, 10, 100, and 1000 *μ*g/mL) of PLE for 24 h. Cell viability was measured by using the EZ-Cytox cell viability assay kit. IC50 values were determined from concentration response curves using Prism 5.0 Software. The IC50 value of BT549 LATS1/2 KO cells was 713 *μ*g/mL. Error bars represent ± SEM from *n* = 3 per group. (b) To measure levels of apoptosis-related protein, western blot was performed on wild-type and LATS1/2 KO BT549 cells treated with PLE at 50 and 100 *μ*g/mL for 12 h. The lysates were immunoblotted with the indicated antibodies.

## References

[1] Yu H., Qiu J. F., Ma L. J., Hu Y. J., Li P., Wan J. B. (2017). Phytochemical and phytopharmacological review of *Perilla frutescens L*. (Labiatae), a traditional edible-medicinal herb in China. *Food and Chemical Toxicology*.

[2] Zhou X. J., Yan L. L., Yin P. P. (2014). Structural characterisation and antioxidant activity evaluation of phenolic compounds from cold-pressed *Perilla frutescens* var. arguta seed flour. *Food Chemistry*.

[3] Asif M. (2012). Phytochemical study of polyphenols in *Perilla Frutescens* as an antioxidant. *Avicenna Journal of Phytomedicine*.

[4] Lim H. J., Woo K. W., Lee K. R., Lee S. K., Kim H. P. (2014). Inhibition of proinflammatory cytokine generation in lung inflammation by the leaves of *perilla frutescens* and its constituents. *Biomolecules Therapeutics (Seoul)*.

[5] Ueda H., Yamazaki M. (2001). Anti-inflammatory and anti-allergic actions by oral administration of a perilla leaf extract in mice. *Bioscience Biotechnology and Biochemistry*.

[6] Makino T., Furuta Y., Wakushima H., Fujii H., Saito K., Kano Y. (2003). Anti-allergic effect of *Perilla frutescens* and its active constituents. *Phytotherapy Research*.

[7] Kwak Y., Ju J. (2015). Inhibitory activities of *Perilla frutescens* britton leaf extract against the growth, migration, and adhesion of human cancer cells. *Nutrition and Research Practice*.

[8] Abd El-Hafeez A. A., Fujimura T., Kamei R. (2017). Synergistic tumor suppression by a Perilla frutescens-derived methoxyflavanone and anti-cancer tyrosine kinase inhibitors in A549 human lung adenocarcinoma. *Cytotechnology*.

[9] Cho J., Tremmel L., Rho O. (2015). Evaluation of pentacyclic triterpenes found in *Perilla frutescens* for inhibition of skin tumor promotion by 12-O-tetradecanoylphorbol-13-acetate. *Oncotarget*.

[10] Ueda H., Yamazaki C., Yamazaki M. (2002). Luteolin as an anti-inflammatory and anti-allergic constituent of *Perilla frutescens*. *Biological and Pharmaceutical Bulletin*.

[11] Banno N., Akihisa T., Tokuda H. (2004). Triterpene acids from the leaves of *Perilla frutescens* and their anti-inflammatory and antitumor-promoting effects. *Bioscience Biotechnology and Biochemistry*.

[12] Dong J., Feldmann G., Huang J. (2007). Elucidation of a universal size-control mechanism in Drosophila and mammals. *Cell*.

[13] Plouffe S. W., Hong A. W., Guan K. L. (2015). Disease implications of the Hippo/YAP pathway. *Trends in Molecular Medicine*.

[14] Yu F. X., Guan K. L. (2013). The Hippo pathway: regulators and regulations. *Genes and Development*.

[15] Hansen C. G., Moroishi T., Guan K. L. (2015). YAP and TAZ: a nexus for Hippo signaling and beyond. *Trends Cell Biology*.

[16] Zhao B., Wei X., Li W. (2007). Inactivation of YAP oncoprotein by the Hippo pathway is involved in cell contact inhibition and tissue growth control. *Genes and Development*.

[17] Overholtzer M., Zhang J., Smolen G. A. (2006). Transforming properties of YAP, a candidate oncogene on the chromosome 11q22 amplicon. *Proceedings of the National Academy of Sciences of the United States of America*.

[18] Vaz-Luis I., Ottesen R. A., Hughes M. E. (2014). Outcomes by tumor subtype and treatment pattern in women with small, node-negative breast cancer: a multi-institutional study. *Journal of Clinical Oncology*.

[19] Shah S. P., Roth A., Goya R. (2012). The clonal and mutational evolution spectrum of primary triple-negative breast cancers. *Nature*.

[20] Collignon J., Lousberg L., Schroeder H., Jerusalem G. (2016). Triple-negative breast cancer: treatment challenges and solutions. *Breast Cancer (Dove Med Press)*.

[21] Zhang J., Yao S., Hu Q. (2016). Genetic variations in the Hippo signaling pathway and breast cancer risk in African American women in the AMBER consortium. *Carcinogenesis*.

[22] Chang S. S., Yamaguchi H., Xia W. (2017). Aurora A kinase activates YAP signaling in triple-negative breast cancer. *Oncogene*.

[23] Guo L., Zheng J., Zhang J., Wang H., Shao G., Teng L. (2016). Knockdown of TAZ modifies triple-negative breast cancer cell sensitivity to EGFR inhibitors by regulating YAP expression. *Oncology Reports*.

[24] Wu L., Yang X. (2018). Targeting the Hippo pathway for breast cancer therapy. *Cancers (Basel)*.

[25] Mo J. S. (2017). The role of extracellular biophysical cues in modulating the Hippo-YAP pathway. *BMB Reports*.

[26] Meng Z., Moroishi T., Mottier-Pavie V. (2015). MAP4K family kinases act in parallel to MST1/2 to activate LATS1/2 in the Hippo pathway. *Nature Communications*.

[27] Mo J. S., Meng Z., Kim Y. C. (2015). Cellular energy stress induces AMPK-mediated regulation of YAP and the Hippo pathway. *Nature Cell Biology*.

[28] Peng Y., Ye J., Kong J. (2005). Determination of phenolic compounds in *Perilla frutescens L*. by capillary electrophoresis with electrochemical detection. *Journal of Agricultural Food Chemistry*.

[29] Zhao B., Li L., Tumaneng K., Wang C. Y., Guan K. L. (2010). A coordinated phosphorylation by Lats and CK1 regulates YAP stability through SCF (beta-TRCP). *Genes and Development*.

[30] Zhao B., Lei Q. Y., Guan K. L. (2008). The Hippo-YAP pathway: new connections between regulation of organ size and cancer. *Current Opinion in Cell Biology*.

[31] Yagi R., Chen L. F., Shigesada K., Murakami Y., Ito Y. (1999). A WW domain-containing yes-associated protein (YAP) is a novel transcriptional co-activator. *EMBO Journal*.

[32] Kim C. L., Choi S. H., Mo J. S. (2019). Role of the Hippo pathway in fibrosis and cancer. *Cells*.

[33] Zhao B., Ye X., Yu J. (2008). TEAD mediates YAP-dependent gene induction and growth control. *Genes and Development*.

[34] Yu F. X., Zhao B., Guan K. L. (2015). Hippo pathway in organ size control, tissue homeostasis, and cancer. *Cell*.

[35] Chan E. H., Nousiainen M., Chalamalasetty R. B., Schafer A., Nigg E. A., Sillje H. H. (2005). The Ste20-like kinase Mst2 activates the human large tumor suppressor kinase Lats1. *Oncogene*.

[36] Glantschnig H., Rodan G. A., Reszka A. A. (2002). Mapping of MST1 kinase sites of phosphorylation. Activation and autophosphorylation. *Journal of Biological Chemistry*.

[37] Praskova M., Khoklatchev A., Ortiz-Vega S., Avruch J. (2004). Regulation of the MST1 kinase by autophosphorylation, by the growth inhibitory proteins, RASSF1 and NORE1, and by Ras. *Biochemical Journal*.

[38] Zheng Y., Wang W., Liu B., Deng H., Uster E., Pan D. (2015). Identification of happyhour/MAP4K as alternative HPO/MST-like kinases in the Hippo kinase cascade. *Development Cell*.

[39] Li S., Cho Y. S., Yue T., Ip Y. T., Jiang J. (2015). Overlapping functions of the MAP4K family kinases Hppy and Msn in Hippo signaling. *Cell Discovery*.

[40] Harvey K. F., Zhang X., Thomas D. M. (2013). The Hippo pathway and human cancer. *Nature Reviews Cancer*.

[41] Mo J. S., Park H. W., Guan K. L. (2014). The Hippo signaling pathway in stem cell biology and cancer. *EMBO Reports*.

[42] Zhang K., Qi H. X., Hu Z. M. (2015). YAP and TAZ take center stage in cancer. *Biochemistry*.

[43] Kaneko K. J., Cullinan E. B., Latham K. E., DePamphilis M. L. (1997). Transcription factor mTEAD-2 is selectively expressed at the beginning of zygotic gene expression in the mouse. *Development*.

[44] Vassilev A., Kaneko K. J., Shu H., Zhao Y., DePamphilis M. L. (2001). TEAD/TEF transcription factors utilize the activation domain of YAP65, a SRC/Yes-associated protein localized in the cytoplasm. *Genes and Development*.

[45] Yockey C. E., Smith G., Izumo S., Shimizu N. (1996). cDNA cloning and characterization of murine transcriptional enhancer factor-1-related protein 1, a transcription factor that binds to the M-CAT motif. *Journal of Biological Chemistry*.

[46] Chan S. W., Lim C. J., Guo K. (2008). A role for TAZ in migration, invasion, and tumorigenesis of breast cancer cells. *Cancer Research*.

[47] Lamar J. M., Stern P., Liu H., Schindler J. W., Jiang Z. G., Hynes R. O. (2012). The Hippo pathway target, YAP, promotes metastasis through its TEAD-interaction domain. *Proceedings of the National Academy of Sciences of the United States of America*.

[48] Torre L. A., Bray F., Siegel R. L., Ferlay J., Lortet-Tieulent J., Jemal A. (2015). Global cancer statistics, 2012. *CA A Cancer Journal for Clinicians*.

[49] Cleator S., Heller W., Coombes R. C. (2007). Triple-negative breast cancer: therapeutic options. *Lancet Oncology*.

[50] Dent R., Trudeau M., Pritchard K. I. (2007). Triple-negative breast cancer: clinical features and patterns of recurrence. *Clinical Cancer Research*.

[51] Camargo F. D., Gokhale S., Johnnidis J. B. (2007). YAP1 increases organ size and expands undifferentiated progenitor cells. *Current Biology*.

[52] Johnson R., Halder G. (2014). The two faces of Hippo: targeting the Hippo pathway for regenerative medicine and cancer treatment. *Nature Reviews Drug Discovery*.

[53] Yimlamai D., Christodoulou C., Galli G. G. (2014). Hippo pathway activity influences liver cell fate. *Cell*.

[54] Cordenonsi M., Zanconato F., Azzolin L. (2011). The Hippo transducer TAZ confers cancer stem cell-related traits on breast cancer cells. *Cell*.

[55] Diaz-Martin J., Lopez-Garcia M. A., Romero-Perez L. (2015). Nuclear TAZ expression associates with the triple-negative phenotype in breast cancer. *Endocrine Related Cancer*.

[56] Li Y. W., Xu J., Zhu G. Y. (2018). Apigenin suppresses the stem cell-like properties of triple-negative breast cancer cells by inhibiting YAP/TAZ activity. *Cell Death Discovery*.

[57] Yuan H., Ma Q., Ye L., Piao G. (2016). The traditional medicine and modern medicine from natural products. *Molecules*.

[58] Ji H. F., Li X. J., Zhang H. Y. (2009). Natural products and drug discovery. can thousands of years of ancient medical knowledge lead us to new and powerful drug combinations in the fight against cancer and dementia?. *EMBO Reports*.

[59] Lin Y., Shi R., Wang X., Shen H. M. (2008). Luteolin, a flavonoid with potential for cancer prevention and therapy. *Current Cancer Drug Targets*.

[60] Lim D. Y., Jeong Y., Tyner A. L., Park J. H. (2007). Induction of cell cycle arrest and apoptosis in HT-29 human colon cancer cells by the dietary compound luteolin. *American Journal of Physiology-Gastrointestinal and Liver Physiology*.

[61] Selvendiran K., Koga H., Ueno T. (2006). Luteolin promotes degradation in signal transducer and activator of transcription 3 in human hepatoma cells: an implication for the antitumor potential of flavonoids. *Cancer Research*.

[62] Osakabe N., Yasuda A., Natsume M., Yoshikawa T. (2004). Rosmarinic acid inhibits epidermal inflammatory responses: anticarcinogenic effect of *Perilla frutescens* extract in the murine two-stage skin model. *Carcinogenesis*.

[63] Hur Y. G., Yun Y., Won J. (2004). Rosmarinic acid induces p56lck-dependent apoptosis in Jurkat and peripheral T cells via mitochondrial pathway independent from FAS/FAS ligand interaction. *Journal of Immunology*.

[64] Moon D. O., Kim M. O., Lee J. D., Choi Y. H., Kim G. Y. (2010). Rosmarinic acid sensitizes cell death through suppression of TNF-alpha-induced NF-kappaB activation and ROS generation in human leukemia U937 cells. *Cancer Letters*.

