# Supplementary material for: Extracts of Perilla frutescens var. Acuta (Odash.) Kudo Leaves Have Antitumor Effects on Breast Cancer Cells by Suppressing YAP Activity
Source: Evid Based Complement Alternat Med. 2021 Feb 15;2021:5619761. doi: 10.1155/2021/5619761 (PMC7899781; doi:10.1155/2021/5619761)
Supplement: Supplementary Materials — Figure S1. Perilla frutescens var. acuta (Odash.) Kudo leaf extract (PLE) induces YAP phosphorylation. PLE increased YAP phosphorylation. HEK293A cells were treated with various natural compounds (50 μg/mL) for 6 h. Dimethyl sulfoxide (DMSO) was used as a control treatment. Phosphorylated proteins were detected using a phos-tag gel. Vinculin was used as the loading control. Figure S2. PLE has no effect on YAP mediated RUNX2-luferase reporter activity. HEK293A cells were co-transfected with a 6 × OSE2-luciferase reporter for RUNX2, with or without HA-YAP2. HEK293A cells were treated with PLE in a concentration-dependent manner. Cells were harvested 10 h after treatment. Representative results of a single experiment with n = 3 biological replicates; three independent experiments were carried out. Luciferase activity was measured and normalized to that of co-transfected Renilla. Figure S3. PLE inhibited mRNA expression of YAP target genes in a MAP4K4/6/7-dependent manner. Wild-type and MAP4K4/6/7 KO HEK293A cells were treated with PLE for 12 h, and then mRNA levels of CTGF and CYR61 were measured using RT-qPCR (error bars represent ± SEM from n = 3 per group). ∗p < 0.05, ∗∗p < 0.01, and ∗∗∗p < 0.001; Student's t-test (unpaired, one-tailed) was used for statistical analysis. Figure S4. Expression of YAP and TAZ on human BC and normal cell lines was analyzed by western blot. Cell lysates were subjected to immunoblotting with the indicated antibodies. Both YAP and TAZ were expressed predominantly in MDA-MB-231 and BT549 human BC cell lines. Figure S5. Effects of PLE on the viability of MCF10A cells. (a) MCF10A cells were treated using the indicated concentrations of PLE for 24 h. Cell viability was measured by using the EZ-Cytox cell viability assay kit. IC50 values were calculated from concentration response curves using Prism 5.0 software. The IC50 value of MCF10A cells was 680.5 μg/mL. Error bars represent ± SEM from n = 3 per group. (b) MCF10A cells were treated with [file 5619761.f1.zip › 5619761-Supplementary Figure 6.pptx]

## Slide 1
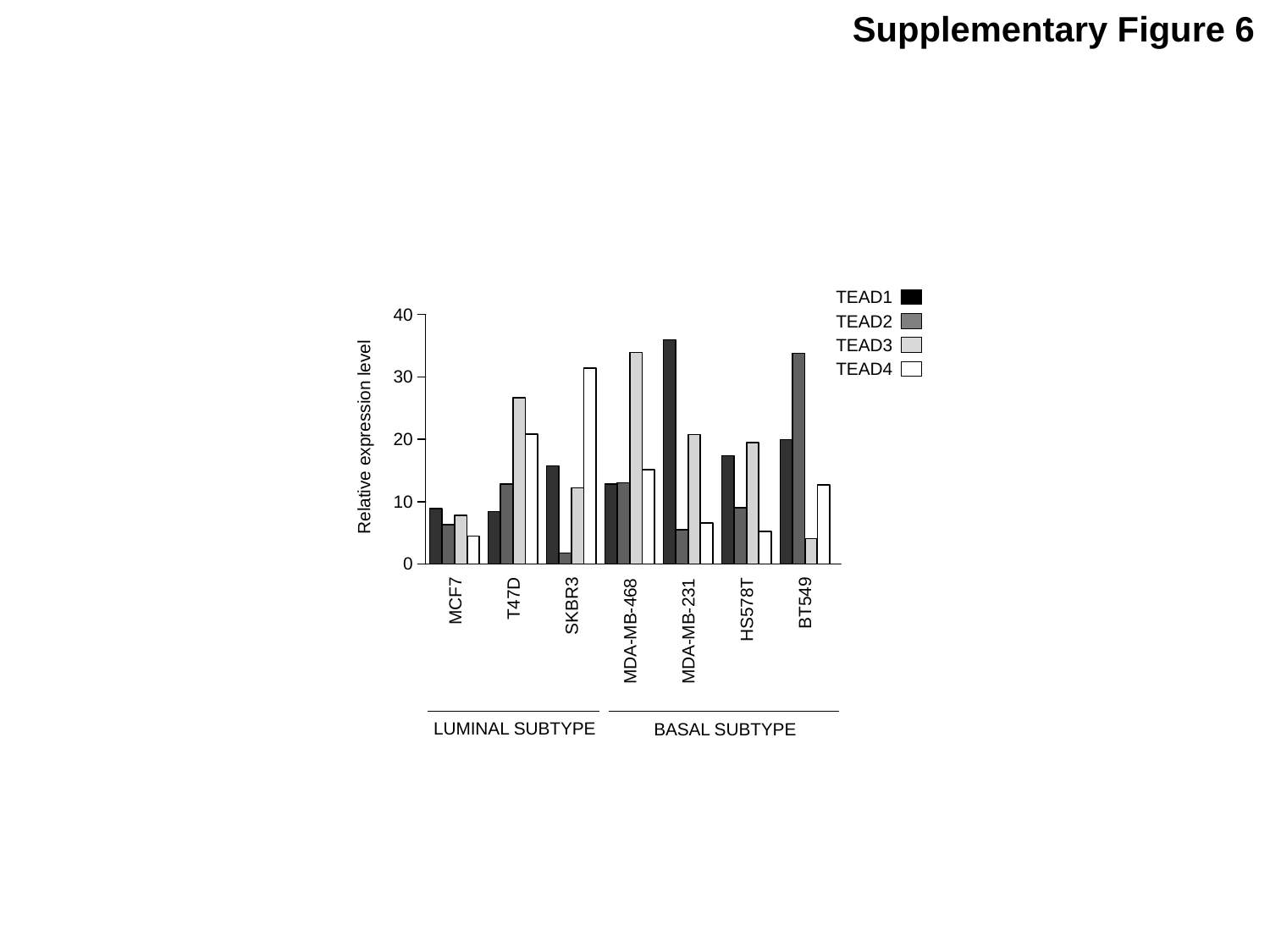

Supplementary Figure 6
TEAD1
40
TEAD2
TEAD3
TEAD4
30
Relative expression level
20
10
0
T47D
MCF7
BT549
SKBR3
HS578T
MDA-MB-468
MDA-MB-231
LUMINAL SUBTYPE
BASAL SUBTYPE
